# A diagnostic clue: New onset alopecia sparing white hairs

**DOI:** 10.1016/j.jdcr.2023.11.030

**Published:** 2023-12-16

**Authors:** Margaret Chen, Lucette Liddell, Robert Brodell

**Affiliations:** Department of Dermatology, University of Mississippi Medical Center, Jackson, Mississippi

**Keywords:** alopecia, alopecia areata, autoimmune, melanocytes, T cell-mediated

## Case description

**Fig 1:**
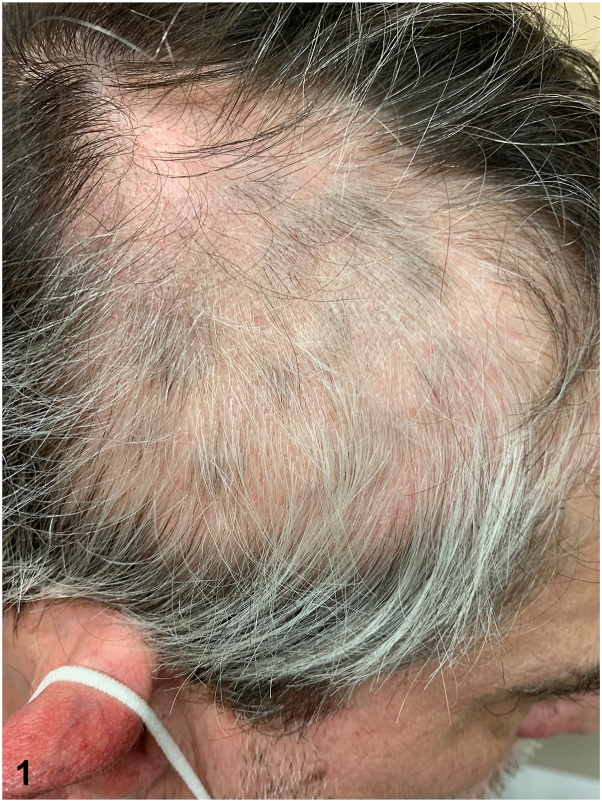

A 55-year-old man presented to dermatology clinic with a smooth alopecic patch on the right temporal scalp of 4 months’ duration. He additionally reported a history of hair loss on the frontal scalp that started 6 months after a left partial hemi-glossectomy for squamous cell carcinoma of the tongue in 2011. Physical exam revealed a 5.5-centimeter circular alopecic patch on the right temporal scalp with white hairs preserved throughout the patch and rare exclamation point hairs around the periphery. Follicular ostia were preserved. Treatment was initiated with 0.6 mL of intralesional triamcinolone acetonide injected intradermally at a concentration of 10 mg/mL on 2 occasions 6 weeks apart. A photo taken 6 weeks after the initial treatment demonstrated regrowth of pigmented hair in small tufts throughout the alopecic patch (Fig 1).

**Question 1: The best initial monotherapy for a solitary 5.5-centimeter patch of alopecia areata on the scalp is?**

A. Topical clobetasol solution, applied twice daily for 6 months
B. Topical pimecrolimus 1% cream, applied twice daily for 6 months
C. Intralesional triamcinolone acetonide, 2.5 to 10 mg/mL, in serial injections every 4 to 6 weeks
D. Oral baricitinib at a dose of 2 milligrams once daily
E. Oral ritlecitinib at a dose of 50 milligrams once daily

**Answers:**

A. Topical clobetasol solution, applied twice daily for 6 months – Incorrect. Clobetasol is a high-potency steroid. Prolonged consistent use can result in atrophy and hypopigmentation of the affected area.
B. Topical pimecrolimus 1% cream, applied twice daily for 6 months – Incorrect. There is no evidence of benefit for topical calcineurin inhibitors in the treatment of alopecia areata.
C. Intralesional triamcinolone acetonide, 2.5 to 10 mg/mL, in serial injections every 4 to 6 weeks – Correct. Intralesional corticosteroids, in a concentration of 2.5 to 10 mg/mL, are considered first-line therapy for localized alopecia areata in adults. A concentration of 10 mg/mL with a maximum total of 2 milliliters has been used in the treatment of alopecia areata in treatments 4 to 6 weeks apart.^1^ Other experts have used concentrations of triamcinolone acetonide in the range of 2.5 to 10 mg/mL, recommending a maximum volume of 3 milliliters on the scalp in 1 visit.^2^
D. Oral baricitinib at a dose of 2 milligrams once daily – Incorrect. Oral baricitinib is an oral Janus kinase inhibitor approved for adults with severe alopecia areata. In clinical trials of baricitinib, patients with severe alopecia areata, defined as 50% or greater scalp hair loss, demonstrated improvement in hair regrowth after taking 2 milligrams of the medicine daily over a period of 36 weeks. This patient had localized, not severe, alopecia areata. In addition, this patient’s history of malignancy may have precluded him from treatment with an oral Janus kinase inhibitor because of its immunosuppressive effects.
E. Oral ritlecitinib at a dose of 50 milligrams once daily – Incorrect. Oral ritlecitinib is an oral Janus kinase inhibitor recently approved for adults and adolescents (age 12 and over) with severe alopecia. Given this patient’s localized disease and history of malignancy, oral ritlecitinib would not be the most appropriate initial therapy.

**Question 2: What pattern of hair regrowth is exhibited in this patient?**

A. Targetoid hair regrowth pattern
B. Perinevoid regrowth pattern
C. Treatment-specific regrowth pattern
D. Renbok phenomenon
E. Castling phenomenon

**Answers:**

A. Targetoid hair regrowth pattern – Incorrect. A targetoid (or concentric) hair regrowth pattern is characterized by concentric zones of hair regrowth with alternating dark hair, white hair, and alopecia bands, creating a polycentric or targetoid appearance. This phenomenon is seen in patients receiving treatment for alopecia areata and is considered a sign of recovery, thought secondary to alternating immunological reactions.^3^
B. Perinevoid regrowth pattern – Incorrect. A perinevoid growth pattern is characterized by hair regrowth around melanocytic lesions. This phenomenon may be seen with treated perinevoid alopecia, a variant of alopecia areata associated with a central pigmented nevus and is thought secondary to an inflammatory response against melanocytes.^4^
C. Treatment-specific regrowth pattern – Correct. Alopecia areata is a nonscarring hair loss that is often associated with canities subita, or the sudden appearance of whitening of the hair “overnight” due to preferential targeting and loss of pigmented hair, with preservation of gray or white hair. It is often the case that hypopigmented hairs emerge first in hair recovery, as observed in this patient.^5^
D. Renbok phenomenon – Incorrect. Also known as the inverse Koebner phenomenon, this pattern designates the regrowth of hair in psoriatic lesions in patients with coexisting alopecia areata and psoriasis.
E. Castling phenomenon – Incorrect. The Castling phenomenon describes a pattern of regrowth that is observed at distant sites or on opposite sides from application of immunotherapy treatment such as with squaric acid or dibutylester, suggesting a systemic action.

**Question 3: What is the molecular basis for a patient’s report of hair turning “white overnight” in this diagnosis?**

A. T cell-mediated autoimmune response
B. Neutrophilic inflammatory response
C. Premature desquamation of the inner root sheath
D. Fungal organisms infiltrating the hair shaft
E. Miniaturization of the terminal hair follicles

**Answers:**

A. T cell-mediated autoimmune response – Correct. Alopecia areata is primarily a cell-mediated autoimmune disease in which autoreactive cytotoxic T cells recognize melanocytes within the hair follicle, leading to preferential shedding of pigmented hairs. The collapse of the immune privilege of the hair follicle is thought to be a significant driver of alopecia areata. However, since no destruction of hair-follicle stem cells occurs, the hair follicle retains its capacity to regenerate.
B. Neutrophilic inflammatory response – Incorrect. A lymphocytic response characterizes alopecia areata with a characteristic “swarm of bees” infiltrate at the follicle. By contrast, a neutrophilic infiltrate may be seen in hair loss secondary to dissecting cellulitis, a scarring alopecia.
C. Premature desquamation of the inner root sheath – Incorrect. Premature desquamation of the inner root sheath with concomitant lymphocytic inflammation leading to rupture of the hair follicle is seen in central centrifugal cicatricial alopecia and other scarring alopecia.
D. Fungal organisms infiltrating the hair shaft – Incorrect. Tinea capitis is a fungal infection of the scalp hair follicles commonly leading to alopecia in affected areas. It is primarily caused by dermatophyte species *Microsporum* and *Trichophyton*. Endothrix fungi invade the hair shaft.
E. Miniaturization of the terminal hair follicles – Incorrect. Androgenetic alopecia is characterized by progressive miniaturization of the terminal hair follicles with eventual conversion of terminal hair to vellus hair. This is a characteristic of androgenetic alopecia.

## Conflicts of interest

None disclosed.
